# Simultaneous Quadriceps Tendon Rupture, Contralateral Patellar Tendon Rupture, and Lateral Meniscus Tear Following a Single Fall: A Case Report

**DOI:** 10.7759/cureus.106395

**Published:** 2026-04-03

**Authors:** Dylan Wood, Gabrielle M Meli, Angel J Gonzalez Saez, Benjamin T Lack, Charles H Hennekens, Gary Schwartz

**Affiliations:** 1 Department of Orthopedic Surgery, Nova Southeastern University Dr. Kiran C. Patel College of Allopathic Medicine, Fort Lauderdale, USA; 2 Department of Orthopedic Surgery, University of Miami Miller School of Medicine, Miami, USA; 3 Department of Orthopedic Surgery, Nova Southeastern University Dr. Kiran C. Patel College of Osteopathic Medicine, Fort Lauderdale, USA; 4 Department of Orthopedic Surgery, Florida Atlantic University Charles E. Schmidt College of Medicine, Boca Raton, USA; 5 Department of Preventative Medicine, Florida Atlantic University Charles E. Schmidt College of Medicine, Boca Raton, USA; 6 Department of Orthopedics, Nova Southeastern University Dr. Kiran C. Patel College of Allopathic Medicine, Fort Lauderdale, USA

**Keywords:** clinical case report, orthopedic trauma, patellar tendon rupture, simultaneous meniscus tear, trauma

## Abstract

Simultaneous ruptures of the quadriceps tendon and contralateral patellar tendon are exceedingly rare, particularly in patients without serious comorbidities or other known predisposing factors. We present the case of an obese 31-year-old man without metabolic syndrome or any other known predisposing risk factors who suffered a right quadriceps tendon rupture and simultaneous left patellar tendon rupture, and tear of the anterior horn of the left lateral meniscus. These injuries occurred while walking downstairs and missing a step. He presented with bilateral knee pain, ecchymosis, and the inability to extend either leg. The diagnosis was confirmed by imaging, and he underwent surgical repairs of both tendons as well as the left lateral meniscus. Thereafter, the postoperative course was uncomplicated. By six months post-surgery, he was able to regain full range of motion in both knees with minimal pain and a normal gait. This case highlights the importance of early recognition, prompt surgical intervention, and structured rehabilitation in achieving optimal recovery in a complex and rare extensor mechanism injury.

## Introduction

Simultaneous tendon ruptures of the quadriceps tendon and contralateral patellar tendon are a rarely encountered injury. To the best of our knowledge, this rare condition has only been reported four times in the peer-reviewed literature [1-4]. Patients with serious comorbidities or other known predisposing factors have increased risks of this rare condition. These serious comorbidities and other known predisposing factors include autoimmune connective tissue disorders, most notably systemic lupus erythematosus, chronic kidney disease, diabetes mellitus, concurrent steroid use, smoking, and the use of fluoroquinolone antibiotics [5,6]. In the absence of any of these comorbid conditions, this simultaneous injury is extremely rare in previously healthy individuals. Age is a risk factor that is positively correlated with quadriceps tendon ruptures but inversely correlated with patellar tendon ruptures [7].

We present the case of a 31-year-old obese man. He had no dyslipidemia, hypertension, or insulin resistance, so he did not have metabolic syndrome. He suffered a right patellar tendon rupture and simultaneous left quadriceps tendon rupture, and tear of the anterior horn of the left lateral meniscus. These injuries occurred while walking downstairs and missing a step. He presented with bilateral knee pain, ecchymosis, and the inability to extend either leg. The diagnosis was confirmed by imaging, and he underwent surgical repairs of both tendons as well as the left lateral meniscus. His postoperative course was relatively uneventful. After physical therapy and a home exercise program, at his six-month visit post operatively he had minimal pain, a normal gait and normal range of motion in both knees. He was recommended to continue his home exercise program and gradually return to his usual daily activities.

## Case presentation

This case involves a 31-year-old man with a height of 175.26 cm and a weight of 122.47 kg. His body mass index was 39.9, which met the requirement for obesity, but did not fulfill the requirements for a diagnosis of metabolic syndrome [8]. The patient had no other known past medical history, family medical history, surgical history, or allergies. He reported a social history of occasional alcohol use.

**Figure 1:**
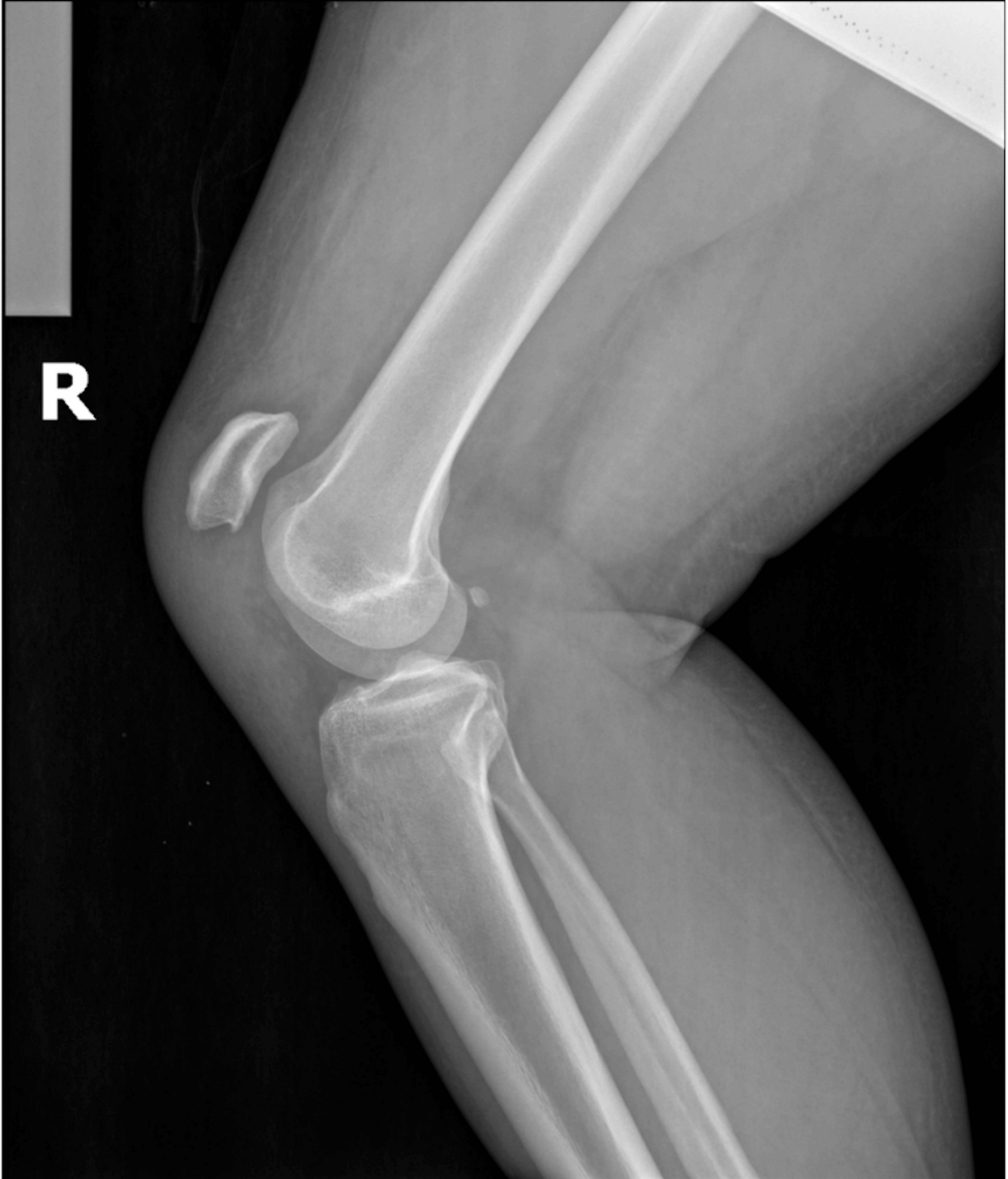
Lateral preoperative radiograph of the right knee Lateral view of the right knee obtiained at presentation demonstrating patella alta, consistent with complete rupture of the patellar tendon. Superior displacement of the patella correlates with the patient’s clinical inability to extend the right knee.

**Figure 2:**
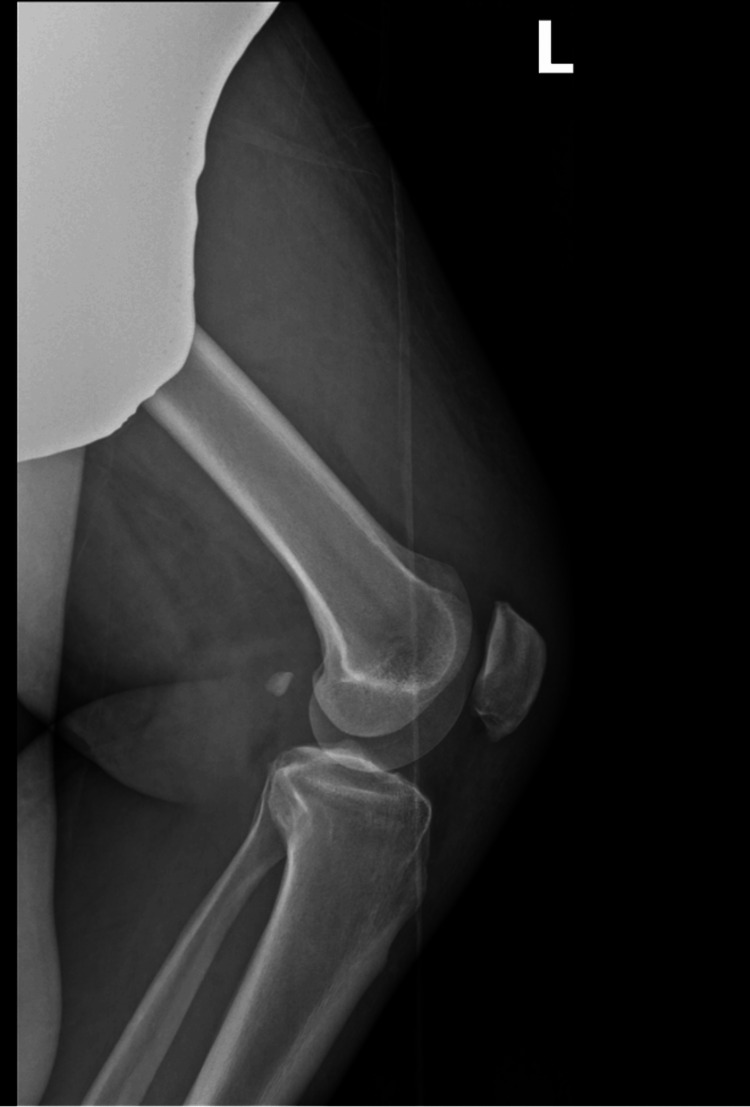
Preoperative lateral radiograph of the left knee Preoperative lateral view of the right knee demonstrating patella baja. This finding is consistent with the rupture of the quadriceps tendon. Inferior displacement of the patella corresponds to the patient's clinical inability to extend his left knee.

**Figure 3:**
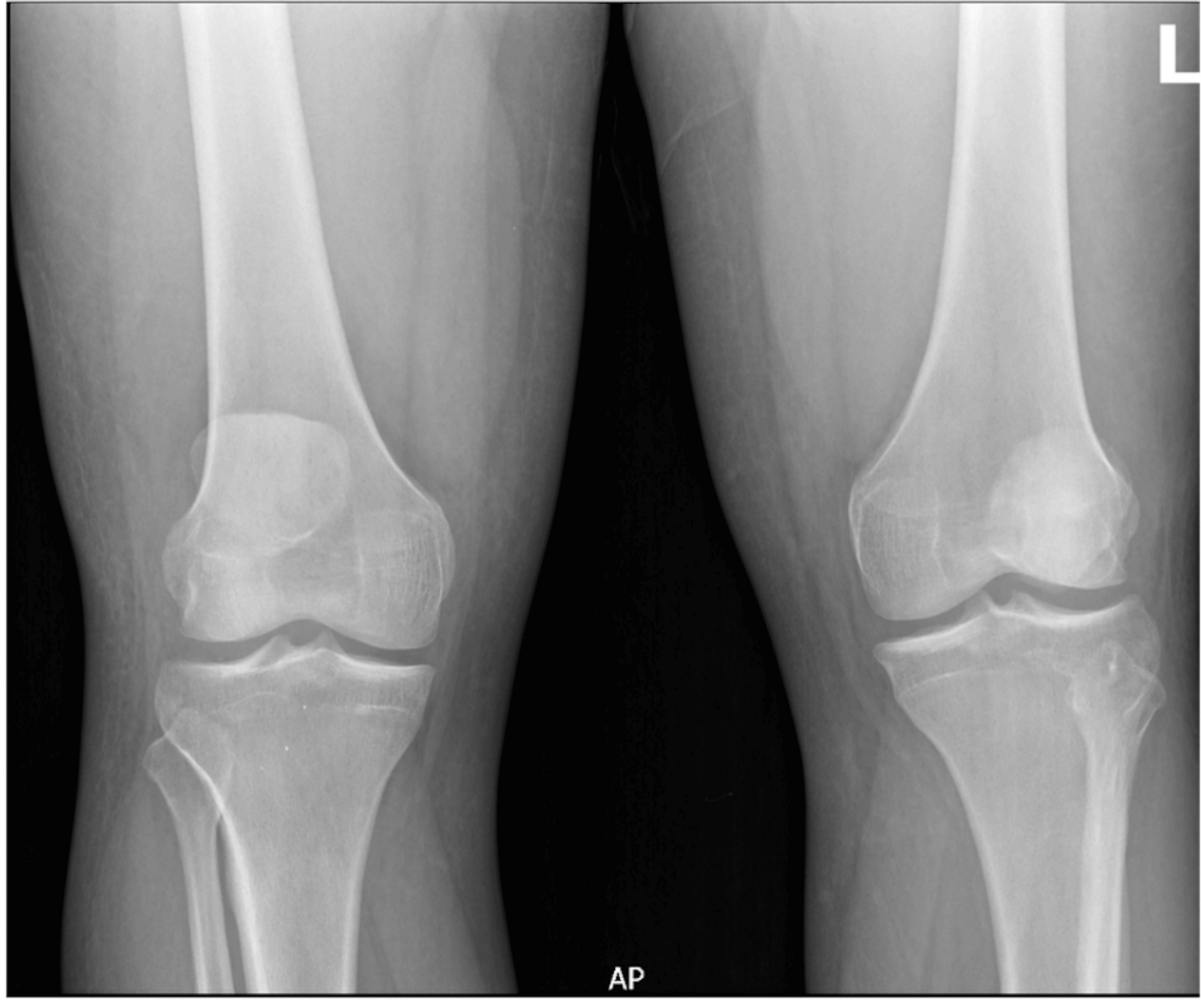
Preoperative AP radiograph of bilateral knees Anteroposterior (AP) radiograph of bilateral knees demonstrating preserved joint spaces with no evidence of a fracture or degenerative changes. Findings are consistent with an extensor mechanism injury.

On September 4, 2024, he presented to an outpatient orthopedic clinic, wheelchair bound with bilateral knee braces, complaining of bilateral knee pain and inability to stand. He stated that on August 20, 2024, he was walking downstairs in his home and missed one step. He stated that he landed on both feet and felt “a pop” in both knees. He presented to an outpatient clinic that day and was diagnosed with a right patellar tendon rupture and left quadriceps tendon rupture. He reported pain and an inability to stand ever since. On physical examination, he had significant bruising and ecchymosis to both knees and an inability to maintain extension of either leg. On the right knee, there was a palpable defect over the patella tendon. On the left knee, there was a palpable defect over the quadriceps tendon. Radiographs were taken demonstrating patella alta of the right knee and patella baja of the left (Figures 1-3).

The patient also underwent an MRI of both knees which revealed a complete tear of the right patella tendon, complete tear of the left quadriceps tendon, and a small, centrally based, white zone tear of the anterior horn of the left lateral meniscus. Due to the severity of the injuries, the patient was recommended a right knee open patellar tendon repair and a left knee open quadriceps tendon repair, with a knee arthroscopy addressing the meniscal pathology.

On September 9, 2024, after a complete preoperative medical evaluation, the patient underwent surgery on both knees. On the right knee, the patient underwent an open transosseous tunnel repair of the right patella tendon using braided polyester sutures, intraarticular and extraarticular repair of the medial and lateral retinaculum, debridement of a tear on the anterior horn of the lateral meniscus, and an intra-articular ropivacaine injection for pain management. On the left knee, the patient underwent an open transosseous tunnel repair of the quadriceps tendon using No. 2 Ethibond braided polyester sutures (Ethicon, Johnson and Johnson, Cincinnati, Ohio, USA), intra-articular and extra-articular repair of the medial and lateral retinaculum, and ropivacaine injection intra-articularly for pain management. Surgical interventions on both knees proceeded without complication. The patient was placed in a dial-lock brace on both knees for post-operative recovery.

**Figure 4:**
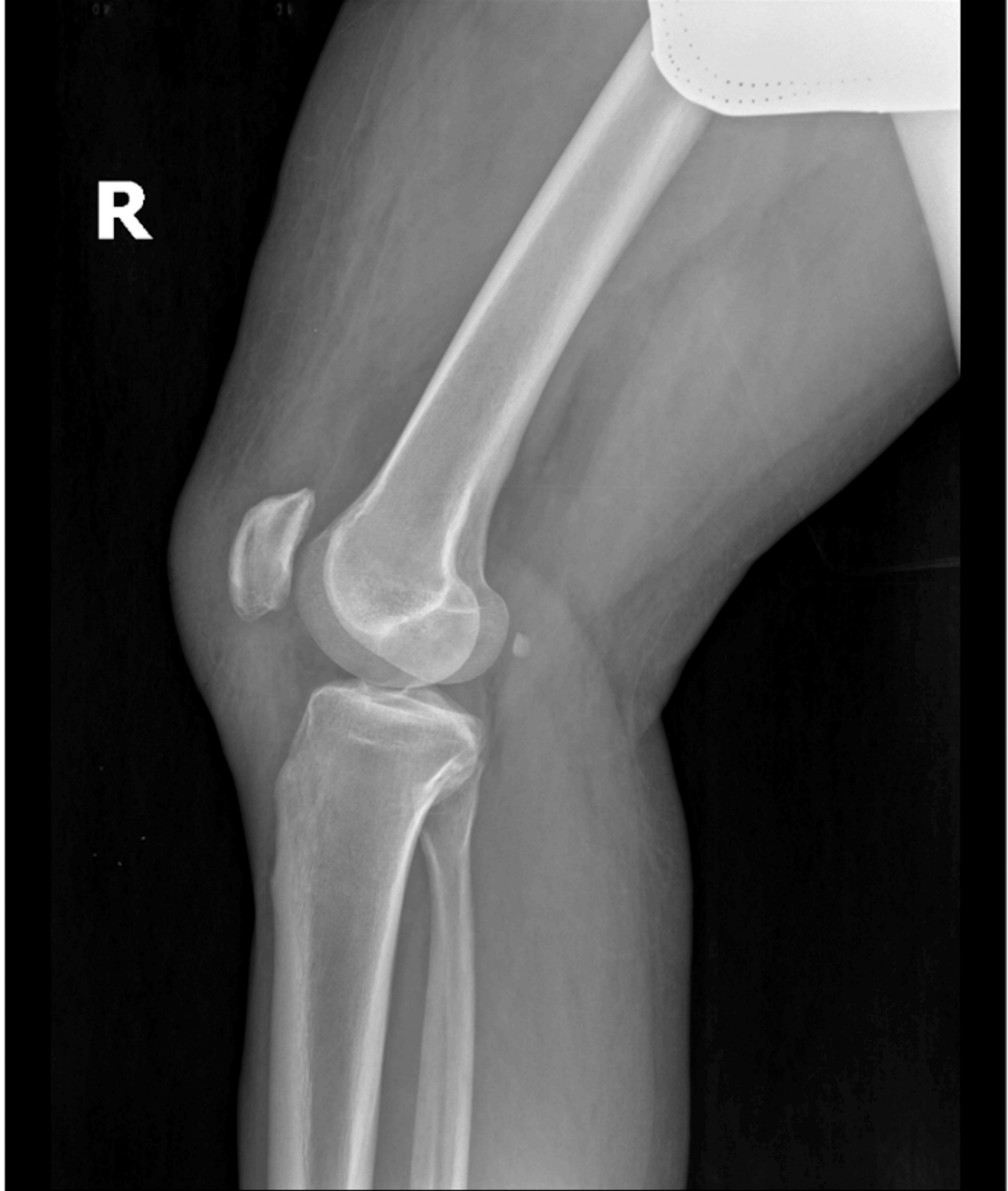
Postoperative lateral radiograph of the right knee Lateral radiograph of the right knee obtained after surgical intervention demonstrates restored patella height and alignment with no hardware or bony abnormalities noted.

**Figure 5:**
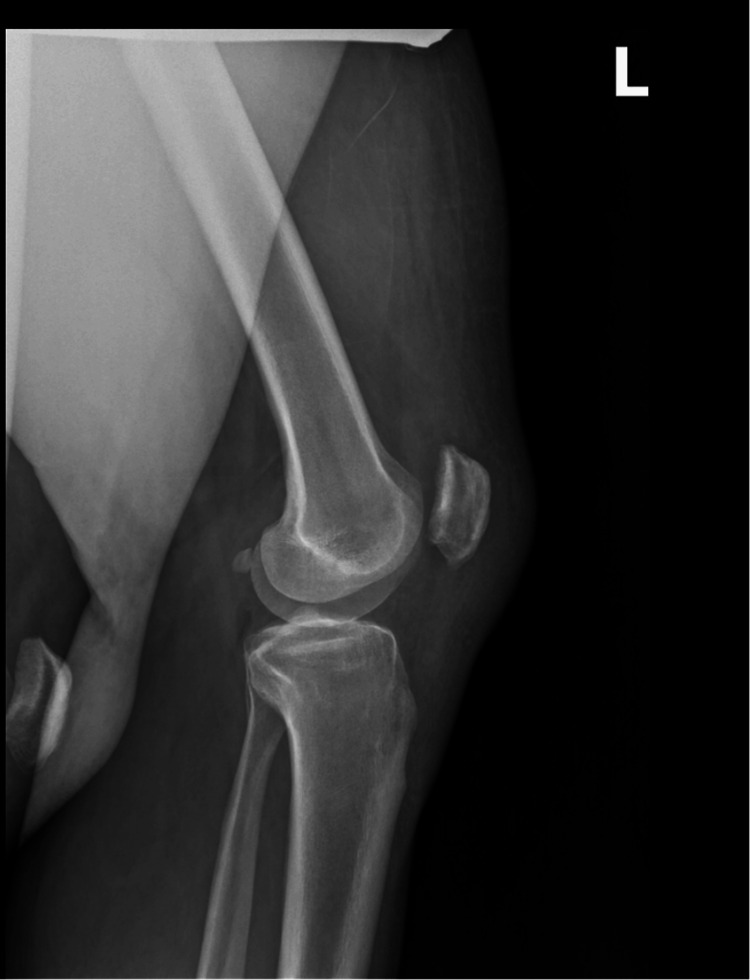
Postoperative lateral radiograph of the left knee Lateral radiograph obtained postoperatively demonstrates restored patellar height and alignment with no hardware or bony abnormalities noted. Findings are consistent with a successful extensor mechanism reconstruction.

**Figure 6:**
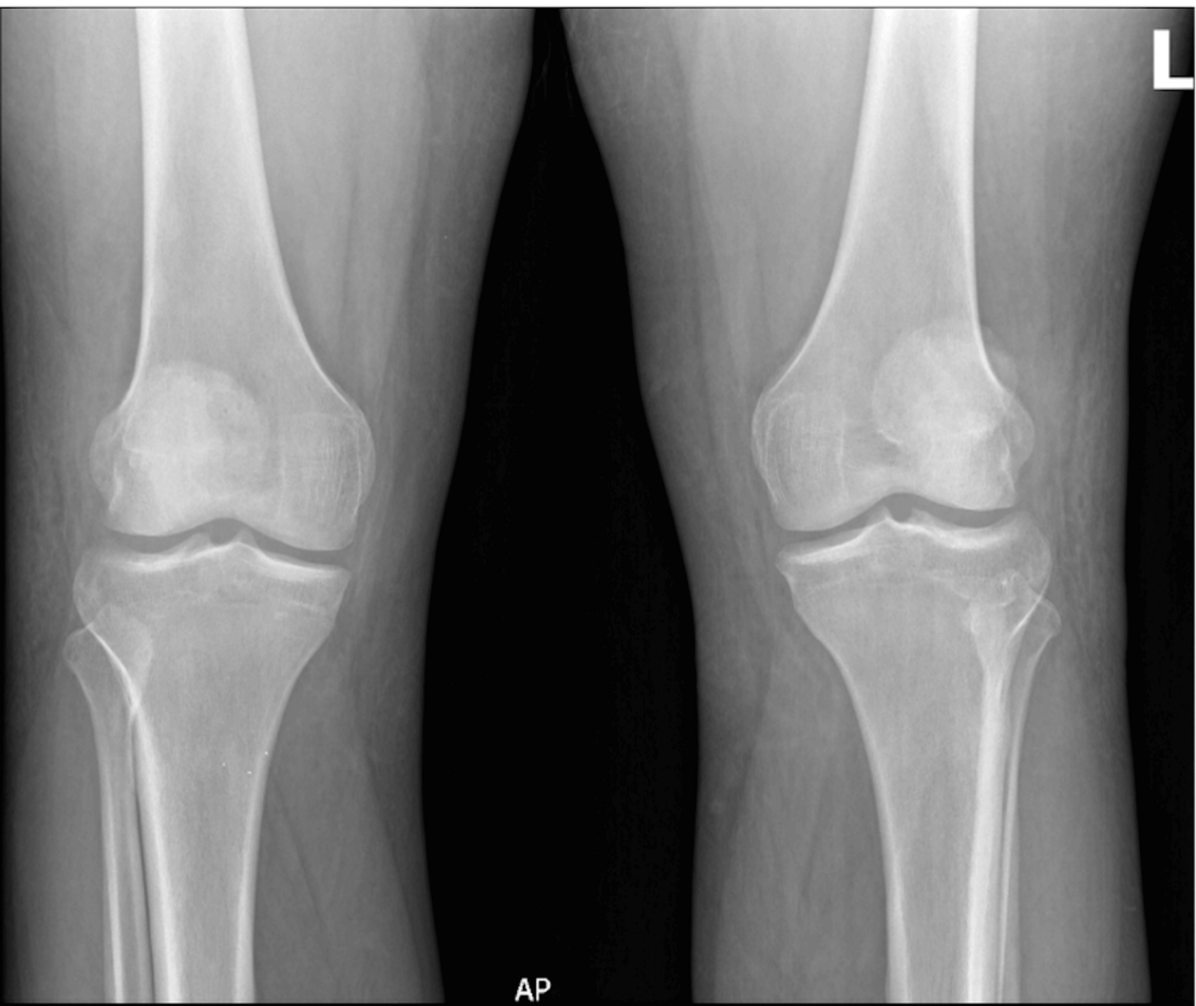
Postoperative anteroposterior (AP) radiograph of bilateral knees AP radiograph demonstrates preserved joint spaces bilaterally with no evidence of fracture or degenerative changes.

The patient was seen again six days postoperatively, revealing dry and clean wounds with no signs of infection, no signs of deep vein thrombosis (DVT), and non-focal neurological examination. Radiographs were taken demonstrating restored patellar height and alignment (Figures 4-6).

The patient was to remain in the dial-lock brace locked at 0° and partial weight-bearing with crutches and was to be re-evaluated one-month post-operatively.

One-month post-operatively, the patient reported one out of 10 pain in his knees. His right knee was able to extend to 0° and flex to 45°; his left knee was able to extend to 0° and flex to 60°. The patient was recommended full weight-bearing and to remain in the dial-lock brace open to 45°, with an increase of 15° per week. The patient was also recommended physical therapy.

On his three-month post-operative visit, the patient reported minimal pain in both knees. He was able to walk with a completely normal gait, extend both knees to 0° and flex both knees to 135°. He was recommended continuation of physical therapy and home exercise programs.

For his six-month post-op visit, he reported minimal pain in both knees. He had a normal gait and range of motion in both knees. He was asked to continue his home exercise program and gradually return to daily activities.

**Figure 7:**
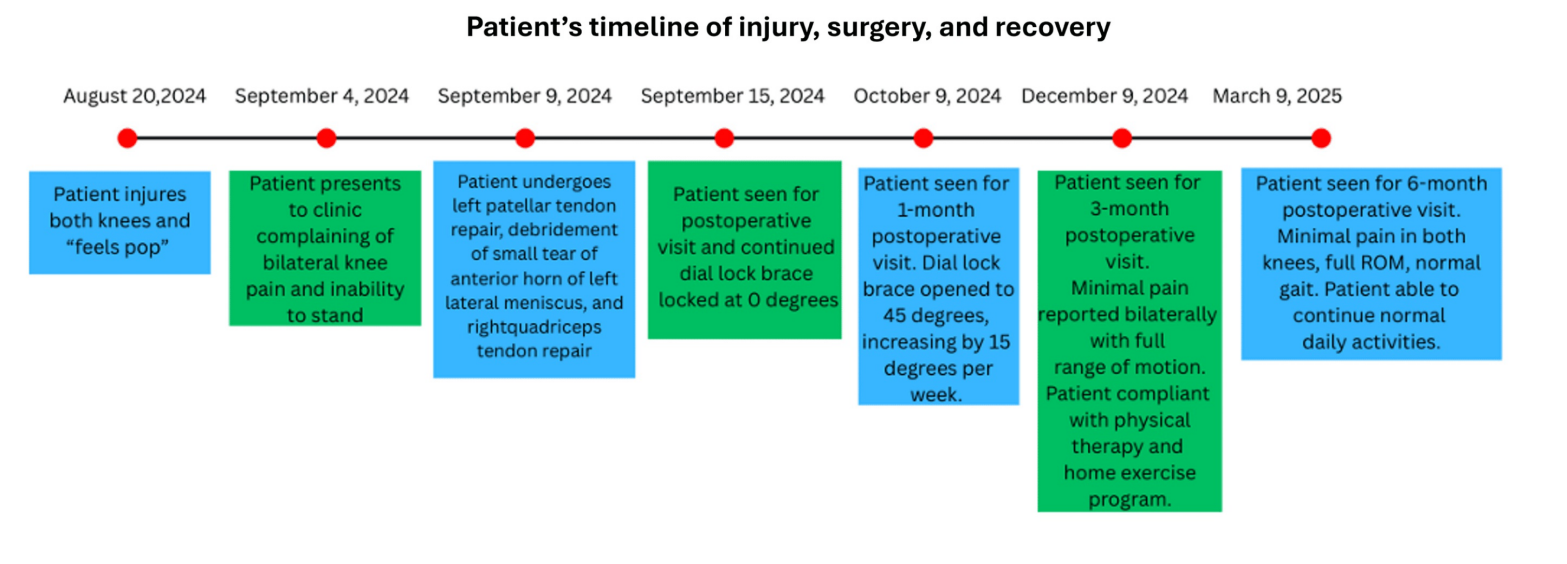
Timeline of the patient's clinical course This timeline shows the sequence of events starting from the initial injury until the six-month postoperative visit. The patient sustained a simultaneous right quadriceps tendon rupture, left patellar tendon rupture, and left lateral meniscus tear with a single fall. He underwent surgical repair of these injuries, followed by postoperative bracing, rehabilitation, and physical therapy. After six months of recovery, the patient demonstrated full range of motion, normal gait, and minimal pain. Image credit: Created by the first author using Microsoft Word (Microsoft Corp., Redmond, WA, USA).

Figure 7 presents a visual timeline of the patient’s injury, surgery, and recovery.

## Discussion

Injuries of the extensor mechanism of the knee are uncommon, with the most common being patella fractures, followed by patella tendon ruptures and quadriceps tendon ruptures. These injuries are diagnosed by the presence of swelling around the knee, a palpable defect, and a positive straight leg test. The diagnosis is confirmed using MRI [6].

The simultaneous rupture of the quadriceps tendon and contralateral patella tendon in an obese man, who was otherwise previously healthy, is an extremely rare combination of severe injuries. Among the many predisposing factors, there are systemic as well as constitutional ones. The systemic factors that predispose patients to simultaneous rupture of the quadriceps tendon and contralateral patella tendon include various rheumatologic diseases, chronic renal failure, and hyperparathyroidism [5]. The strongest constitutional factor is age. Patellar tendon ruptures are more common in patients under 40 years of age, while quadriceps tendon ruptures are more common in patients over 40 years of age [7].

To the best of our knowledge, there are only four other cases reported in the literature of the simultaneous rupture of the patella tendon and quadriceps tendon in a previously healthy individual [1-4] and no others with a lateral meniscus tear.

This patient received a prompt diagnosis and then underwent urgent operative repair. After a rehabilitation period that included crutch-assisted ambulation and a hinged knee brace locked in full extension (0°) for one month, followed by progressive range-of-motion advancement starting at 0-45° for one week and increasing by 15° weekly, the patient regained full knee extension bilaterally and was able to ambulate with a normal gait. This outcome is consistent with prior case reports of successful recovery after prompt surgical intervention [1-4,9]. Systemic reviews further emphasize the importance of early repair for optimal functional outcomes [10,11]. Recent studies suggest no significant difference in outcomes between transosseous tunnel and suture anchor techniques, but highlight the importance of early mobilization [9]. Similarly, in patellar tendon ruptures, early repair is associated with favorable recovery, although rehabilitation protocols vary [10].

This case highlights the importance of urgent surgical intervention followed by an immobilization period and physical therapy for this rare injury [12-14].

## Conclusions

A simultaneous rupture of the quadriceps tendon and a contralateral patellar tendon is a rare injury pattern. It is important to understand the predisposing factors, such as obesity, autoimmune disorders, and kidney disease, as well as other comorbidities, which may be associated with these diagnoses. The mechanism of injury, an eccentric contraction, can result in both injuries. Prompt diagnosis and treatment are important, even though in our case, the definitive treatment occurred at day 20 after the injury. Postoperative treatment, including physical therapy, is extremely important for obtaining the maximum benefit. The surgical techniques used to accomplish the repair may be surgeon-specific. When all the above factors are considered collectively, this injury pattern is readily identifiable.
